# Refining dosing by oral gavage in the dog: A protocol to harmonise welfare

**DOI:** 10.1016/j.vascn.2014.12.007

**Published:** 2015

**Authors:** Laura E. Hall, Sally Robinson, Hannah M. Buchanan-Smith

**Affiliations:** a Behaviour and Evolution Research Group, Psychology, School of Natural Sciences, University of Stirling, Stirling, UK; b Drug Safety and Metabolism, AstraZeneca, Alderley Park, Macclesfield, UK

**Keywords:** Dog, Dosing, Oral, Gavage methods, Toxicology, Refinement, 3Rs, Welfare

## Abstract

**Introduction:**

The dog is a frequently-used, non-rodent species in the safety assessment of new chemical entities. We have a scientific and ethical obligation to ensure that the best quality of data is achieved from their use. Oral gavage is a technique frequently used to deliver a compound directly into the stomach. As with other animals, in the dog, gavage is aversive and the frequency of its use is a cause for welfare concern but little research has been published on the technique nor how to Refine it. A Welfare Assessment Framework (Hall, 2014) was previously developed for use with the laboratory-housed dog and a contrasting pattern of behaviour, cardiovascular and affective measures were found in dogs with positive and negative welfare.

**Methods:**

Using the framework, this study compared the effects of sham dosing (used to attempt to habituate dogs to dosing) and a Refined training protocol against a control, no-training group to determine the benefit to welfare and scientific output of each technique.

**Results:**

Our findings show that sham dosing is ineffective as a habituation technique and ‘primes’ rather than desensitises dogs to dosing. Dogs in the control group showed few changes in parameters across the duration of the study, with some undesirable changes during dosing, while dogs in the Refined treatment group showed improvements in many parameters.

**Discussion:**

It is recommended that if there is no time allocated for pre-study training a no-sham dosing protocol is used. However, brief training periods show a considerable benefit for welfare and quality of data to be obtained from the dogs' use.

## 1 Introduction

### 1.1 The 3Rs and toxicology

There are good reasons why positive welfare should lead to good quality of data output in laboratory-housed animals (see Poole, 1997, for review). Improvements in welfare, which has been defined as “an individual's state in relation to its attempts to cope with its environment” (Broom, 1986), have shown corresponding improvements in data output (as measured by repeatability, sensitivity and validity) in species from mice (Wurbel, 2001) to macaques (Tasker, 2012). Such research has been largely lacking in the dog, with some exceptions (e.g. Hubrecht & Serpell, 1993).

The guiding principles of humane research with animals are the 3Rs: Replacement, Reduction and Refinement (Russell & Burch, 1959). The dog is a common non-rodent model in safety assessment and other research, with > 3200 dogs used in the UK (Home Office, 2013) and > 72,000 used in the USA (USDA, 2014) in 2012. We have an obligation to ensure that the use of the dog is Refined where its use in toxicology cannot be Replaced or Reduced. Refinement is defined as “any approach which avoids or minimises the actual or potential pain, distress and other adverse effects experienced at any time during the life of the animals involved, and which enhances their wellbeing” (Buchanan-Smith et al., 2005, p.381). Our recent research (Hall, 2014) has led to the development of a framework used to identify dogs with negative welfare and producing lower quality data (defined as reduced sensitivity and repeatability, and increased unwanted variation). Another application of this framework is to monitor the effects of planned Refinements and provide empirical evidence for the implementation of changes to housing, husbandry and regulated procedures. The physical and behavioural effects of stress introduced by a dosing technique are undesirable for ethical and scientific reasons.

### 1.2 Oral gavage as a dosing technique

Oral gavage is a technique for delivering a substance directly into the stomach and is frequently used to administer test compounds in research and toxicity testing. It is recognised as an invasive and aversive event in the life of a laboratory animal (Wallace, Sanford, Smith, & Spencer, 1990), and therefore a negative impact on welfare and quality of data output. In a standard one- or three-month toxicology study, dogs may experience daily oral gavage, while other study types may require multiple doses in a day. We estimate that most laboratory-housed dogs which are subject to regulated procedures will experience oral gavage, and the potential impact of oral gavage on welfare may be significant given the regularity of its use. While it is recommended that dogs are introduced to the technique and habituated (commonly referred to as Sham Dosing, ShD) before a study begins (Prescott et al., 2004), there is little standardisation in the method for doing this.

In addition, there is no robust scientific evidence demonstrating a welfare benefit from the procedure of ShD. Apparent cooperation may be a ‘freezing’ response to fear. A proficient technician is able to deliver a dose of a compound quickly and without physical trauma. However, a technique which is invasive, which happens at potentially unpredictable intervals, and is beyond the control of the dog always has the potential to be highly aversive (Laule, 2010). It is unclear whether the practice of ShD has any welfare benefit, although it is widely used.

There is comparatively little guidance published on training of the laboratory-housed dog for procedures (i.e. organisations such as NC3Rs and IAT produce guidance for procedures in rodents) and almost nothing specifically for the Refinement of oral gavage in the dog. However, there is a wealth of literature available (Laule, 2010; McKinley, Buchanan-Smith, Bassett, & Morris, 2003; Prescott, Buchanan-Smith, & Rennie, 2005) supporting the benefits of positive reinforcement training (PRT) for various aspects of husbandry and procedures for many species in the laboratory environment. PRT is also used extensively in the training of dogs in other situations (e.g. (Hiby, Rooney, & Bradshaw, 2004; Batt, Batt, Baguley, & McGreevy, 2008; Fjellanger, Andersen, & McLean, 2002), pet, guide and sniffer dog respectively).

Our previous research using other groups of dogs in the same facility identified convergent validity in patterns of behaviours, cardiovascular parameters, affective state (free-floating mood states, not directed at an object, requiring a lesser degree of information processing, as determined by cognitive bias testing Paul, Harding, and Mendl (2005)) and mechanical pressure threshold (MPT). These factors distinguished welfare states between dogs (see Hall, 2014). Those with more negative welfare showed higher levels of undesirable behaviours (and often more ‘reactive’ behaviours) at baseline in the home pen and in response to behavioural challenges. Dogs also had higher blood pressure at baseline, exhibited a greater cardiovascular response to a brief physical restraint on the procedure table, exhibited a negative affective state and had a lower threshold for mechanical pressure. It is likely that these dogs adapt less well to aversive techniques such as gavage. Anecdotally, technical staff report that some dogs in any study will consistently fail to adapt, which is likely to produce unwanted variation and lower quality data output. This is concerning given the numbers of dogs subject to oral gavage. Understanding the link between positive welfare and high quality of data output is critical for ethical and scientific reasons. The framework is designed to identify those dogs most at risk of negative welfare and highlights the need for harmonisation of training and desensitisation.

The response to a brief physical restraint by a handler (on the procedure table, mimicking that used in regulated procedures) highlighted it as an aspect of study protocol particularly in need of Refinement. This was due to the undesirable changes in behavioural and cardiovascular parameters seen in the absence of a regulated procedure.

### 1.3 Training for procedures through habituation, desensitisation, predictability and control

While habituation may be the most common form of training for aversive events such as restraint, desensitisation is more desirable when positive welfare is to be promoted. Habituation is the process by which the response to a stimulus diminishes by repeated exposure to the stimulus, while desensitisation is the process of reducing the response to an aversive stimulus by pairing a reward (usually food) with the presentation of the stimulus (Laule, 2010). Habituation may be common practice for regulated procedures in a laboratory setting and may result in a decreased behavioural response to the aversive stimulus or event. However, this may not represent actual habituation but rather a “freezing” response and cooperation, while internal arousal has not decreased (e.g. Ruys, Mendoza, Capitanio, & Mason, 2004). It is commonly recommended that some form of “habituation” take place before a study (e.g. Laule, 2010), however the interpretation of its use varies, and there is currently no standardisation in the use of desensitisation within the laboratory environment for the dog (Prescott et al., 2004). Sham dosing (dosing with no compound administered) twice before a study begins is, in our experience, the most common form of habituation used for oral gavage.

Desensitisation or PRT may not be implemented in the laboratory environment because of a lack of understanding of the methodology or benefits of the techniques. Additionally, PRT usually involves giving a food reward which is perceived as undesirable and a source of unwanted variation in safety assessment. The interaction between perceived non-standardised food and the test substance is commonly given as the reason for not standardising desensitisation in the laboratory setting. Instead, negative reinforcement training (NRT) is more commonly used than PRT. NRT is by definition the removal of a stimulus to increase the expression of a behaviour (animal removed from stimulus upon compliance), however in practice it often involves the use of an unpleasant stimulus and as such instils fear, resistance and avoidance (“priming” a strongly negative response to the event), all of which are undesirable states in an in vivo model of a healthy human.

As PRT is likely to have a more positive impact on welfare than NRT, and is also likely to increase rather than decrease cooperation, it should be the preferred training method in the laboratory environment. PRT also increases the animal's ability to control its environment (Bassett & Buchanan-Smith, 2007).

Overmier, Patterson, and Wielkiewicz (1980) found that this ability to exert control increases the positive effects and decreases the negative effects of an event. Therefore, control may reduce the negative effects of an aversive event. Control and predictability are also interlinked, as increased control leads to increased predictability over the occurrence of an event, while increased predictability can lead to an increased ability to exert control, although some aversive events may never be controllable. For a review of the benefits of predictability and perceived control, see Bassett and Buchanan-Smith (2007). A combination of desensitisation, PRT, control and predictability provides a robust method of mitigating the effects of aversive events.

### 1.4 Aims

The first aim of this study was to compare the current sham dosing procedure (ShD group) with a group receiving no sham dosing (Control group) to determine whether the sham dosing procedure alone has a benefit for the dogs' welfare. The second aim was to compare both of these groups with a third group receiving Refined desensitisation and handling (RP group) to determine whether additional training and Refinements to the sham dosing technique have any benefit to dogs' welfare and quality of scientific output.

## 2 Methodology

### 2.1 Overview of study design

Table 1 illustrates the treatment given to each of the three groups in each aspect of the study. There were three phases to the study: Training, Sham Dosing and Dosing. Each of the three groups received different training in the Training and Sham Dosing phases, while treatment was identical for all dogs during the Dosing phase.

The study was subject to a Good Statistical Practice (GSP) review prior to commencing, as per current practice (Peers, South, Ceuppens, Bright, & Pilling, 2014). The following principles of GSP were applied: a parallel group design was used, with a control group which could be compared to the ShD and RP groups; appropriate data analysis was planned before the study began (see Section 2.8); animal numbers were based on previous similar research (Hall, 2014); animals randomised to groups based on age and body weight; and during the treatment phase, the order of dosing was pseudorandomised, taking one dog from each group and repeating. It was not possible to blind technicians or handlers to treatment condition. However, an observer blind to condition also coded behavioural observations and inter-observer reliability was calculated with the authors' observations as 0.8 (of maximum 1).

### 2.2 Subjects

Each of the three groups consisted of six naïve female Alderley Park strain beagles (age range 21–25 months). Dogs were selected as a convenience sample, with insufficient male dogs being available to match the number of female dogs. Our previous research (Hall, 2014) found that behaviour and welfare in the laboratory environment did not vary significantly by sex. Dogs were assigned to groups by weight, age, and siblings were dispersed throughout groups. Temperament was assessed using the behavioural measures described in Hall (2014), and dogs with similar patterns of behaviour were dispersed throughout the three groups.

Dogs were group-housed in three interlinked home pens (of 2.5 m^2^ each) per group, with single-housing occurring immediately before and after sham dosing and dosing only. Other than during single-housing, dogs had continuous access to plastic chew toys and an indoor play area containing climbing frames and additional chew toys. Daily husbandry was conducted at 7am and 3pm by the responsible technician. All dogs had received weekly health checks and basic habituation while held as stock, but no structured programme of training.

### 2.3 Behavioural observations

Home pen observations were made before and after training, sham dosing and dosing sessions. Each home pen observation was of five minutes duration. This allowed comparisons between- and within-groups to be made across the three phases (Training, ShD and Dosing), and also before and after dosing sessions. Behavioural observations were also conducted during all training or dosing sessions. All behaviour was recorded on a camcorder and scored remotely using The Observer 10.5XT (Noldus). Home pen behaviour was scored using a combination of instantaneous (30 second intervals) and continuous sampling. Behavioural states are presented as a percentage of time, while behavioural events are presented as a rate per hour. Behaviour during dosing was recorded using continuous sampling only due to short durations (see Martin & Bateson, 2007, for a discussion of measuring behaviour).

### 2.4 Training phase (days 1–9)

During Training phase, dogs in control and ShD groups received no interventions other than a weekly health check. Dogs in RP group received a number of additional Refinements (e.g. PRT, changes to handling and sham dosing technique, increased predictability) as detailed in Appendix A and Appendix B.

### 2.5 Sham Dosing phase (days 10–14)

Two sham doses were delivered to both ShD and RP groups on consecutive days in this phase, following the company protocol. Dogs were taken one at a time, in a pre-determined order, to the procedure pod nearest the pen; once positioned on the table and restrained by the handler, the technician inserted the gavage tube. Dogs were immediately returned to the home pen and allowed to return to group housing once the last dog had received its sham dose.

Dogs in ShD group underwent the standard protocol in which the tube was dipped in warm water before insertion. Dogs in RP group underwent a Refined protocol in which the tube was also coated in palatable paste (Beaphar® Vitamin Malt Paste) and dogs were rewarded with a food treat immediately afterwards. This was to desensitise dogs to the gavage procedure.

### 2.6 Dosing phase (15–19)

On each day of Dosing phase, all groups were dosed with vehicle hydroxypropyl methylcellulose (HPMC, at 2 ml kg^1^) as per the dosing protocol of a standard toxicology study. Dogs in control and ShD groups underwent the identical treatment, while dogs in RP group also continued to receive Refined handling and the predictable signal as detailed in Table 1. No additional food treats were administered to RP group during Dosing phase as it was deemed unlikely that the use of food treats at the time of dosing could be incorporated into Good Laboratory Practice studies.

### 2.7 Other measures

#### 2.7.1 Welfare Monitoring Tool

The WMT was employed to determine whether changes in welfare could be detected by the technician in a manner practical to use in the busy laboratory environment. Behaviours measured included resting, alert, amicable and interactive behaviours, postures and stereotypic behavioural events. Behaviour was scored by the technician for each dog hourly between 8am–3pm, with the exception of 1pm which was during feeding time. Additional behaviours were scored where dosing or sham dosing took place in any given hour. The tool was weighted such that a low score would represent positive welfare, with increasing scores representing decreasing welfare. Results can be found in Section 3.3.

#### 2.7.2 Body weight and food consumption

Food consumption was measured daily throughout the study. Dogs were restricted to single-housing for a two-hour period, during which they were provided with a bowl containing 300 g of SDS standard dog diet. Each bowl was weighed following this period, and the amount in grams which had been eaten recorded. Food consumption data were not available for the week before the study began.

Body weight was measured once weekly, including the week before the study began, giving a total of four readings for each dog. Dogs were individually removed to the weighing scales once weekly by the technician, and their weight in kilograms recorded.

#### 2.7.3 MPT

MPT was conducted twice during the Training phase and three times during the Dosing phase of the study. Dogs were placed unrestrained on the floor of the procedure pod and readings taken with the algometer (TopCat Metrology ‘Prod’) applied to the mid-back of the dog. Three readings were taken on each occasion and the mean calculated. MPT readings taken in ShD and Dosing phases allow a comparison of MPT change as a result of dosing. Three readings were taken on each day, with the mean calculated from these readings. Results are presented in Section 3.4.2.

#### 2.7.4 Time required to dose

One of the aims of improving the behaviour and cooperation of the dogs during dosing was to improve the ease and speed of protocol for the technician. As such, the time taken to dose each dog was measured during each dose. Timing began when the dog was placed on the table and stopped when the dog was removed following dosing.

### 2.8 Data analysis

#### 2.8.1 Behavioural observations

Behavioural states are presented as a percentage of time and behavioural events as a rate per hour. Data which were not normally distributed were transformed using an angular transformation.

All data were entered into SPSS 19.0 for Windows. Normally-distributed data were analysed using factorial ANOVAs (with between-subjects factor of ‘group’ and within-subjects factor of ‘phase’) and planned post-hoc paired-sample (within-subjects) or independent samples (between-subjects) t-tests. Data which were not normally distributed were analysed using Kruskal–Wallis tests and planned post-hoc Mann–Whitney U tests. Results were considered to be significant at p < 0.05 and highly significant at p < 0.001.

### 2.9 Ethical statement

All aspects of this study were conducted in compliance with A(SP)A (1986, updated 2012). Ethical approval was also granted by the Ethics Review Committee of Psychology, University of Stirling and an Ethics Review Panel at AstraZeneca prior to the study beginning.

## 3 Results and interpretation

The results of analysis of home pen behaviour, behaviour during dosing, the welfare monitoring tool and quality of data output are presented in this section. Significant differences (p < 0.05) are indicated by brackets within graphs.

### 3.1 Home pen behaviour

Home pen behaviour was analysed within each phase of the study for between-group differences.

#### 3.1.1 Training phase

**Fig. 1:**
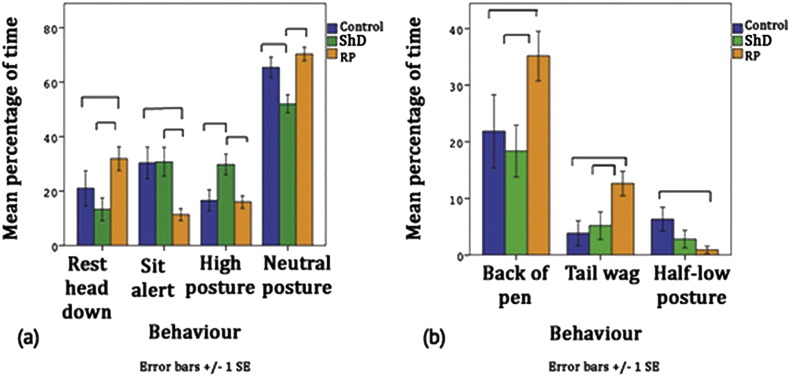
The significant between-group differences in home pen behaviour during Training phase. Lines show significant differences (p < 0.05).

The positive impact of training on RP group is clear during Training phase (Fig. 1). Greater time spent in indicators of positive welfare (resting, neutral posture, back of pen, see Table 2) is seen more in RP group than the others. Control and ShD groups spent more time sitting alert or with high or half-low posture (negative welfare indicators, see Tables 2 and 3).

#### 3.1.2 Dosing phase

**Fig. 2:**
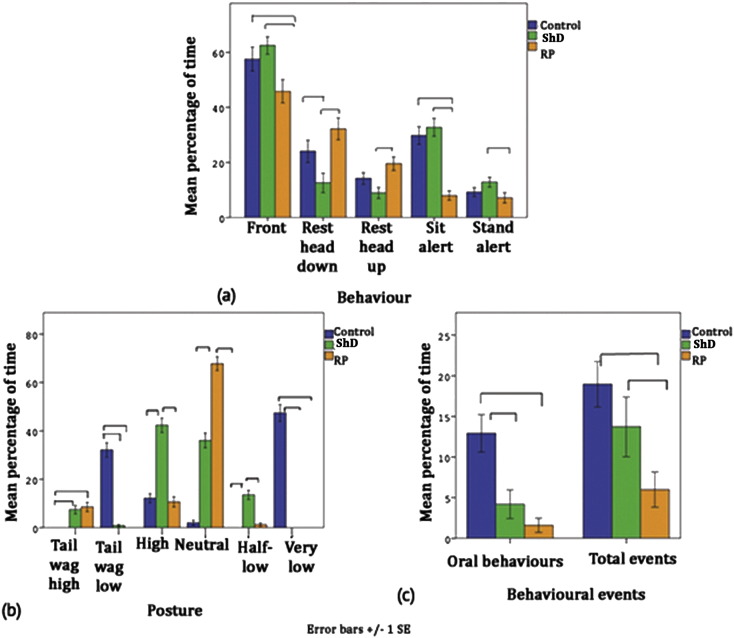
Between-group differences in (a) behavioural states, (b) posture and (c) behavioural events in the home pen during Dosing phase. Lines show significant differences (p < 0.05).

In Dosing phase, RP group continue to exhibit more positive welfare indicators (neutral posture, resting head up or down, see Fig. 2 and Table 4). A difference in welfare-indicating behaviours is seen between the three groups. ShD group demonstrated more negative welfare indicators than control or RP groups (low posture, alert, see Tables 4 and 5).

#### 3.1.3 Differences across phases

Behaviour showed a pattern of greater positive welfare indicators in Training phase (neutral posture, play, see Table 6); while negative welfare indicators (half-low posture, behavioural events, see Table 7) increased across ShD and Dosing phases, as would be expected in response to aversive events. This was consistent across groups, suggesting that throughout the study, welfare was greatest in RP group and lowest in ShD group.

### 3.2 Behaviour during Dosing

There is a clear pattern in behaviour during dosing, with RP showing greater positive welfare indicators (interacting with handler, sit relaxed, neutral posture, see Tables 8 and 9) than control or ShD groups. ShD group also show greater negative welfare indicators than control group (crouching, escape attempts, see Table 8).

### 3.3 Welfare monitoring tool (WMT) scores

There was a significant effect of group on score (F(2, 177) = 5.789, p = 0.004), with ShD group having the highest and RP group the lowest scores. There was no interaction between group and phase (p = 0.5), suggesting that this pattern of higher scores in ShD group was maintained across all phases. Scores also increased for all dogs on the first day of dosing, and for ShD and RP groups during ShD phase, suggesting that these were the most distressing doses.

### 3.4 Quality of data output

#### 3.4.1 Body weight and food consumption

A repeated-measures ANOVA was conducted with one within-subjects factor of Week (4 levels) and one between-subjects factors of group (3 levels). There was no significant effect of Week (p = 0.532), nor a significant interaction between group and Week (p = 0.397). Body weight was very stable across the study regardless of group. Similarly, food consumption was very stable for all dogs across the study, with no effect of phase found (F(2, 321) = 1.691, p = 0.186), although control group dogs had the highest food consumption throughout the study (F(2, 321) = 18.434, p = < 0.001).

#### 3.4.2 MPT testing

There was a significant interaction between group and phase, F(8, 160) = 4.589, p < .001 (Fig. 4). This is due to RP group showing no change over phases (p = .149), while control group (F(4, 20) = 10.17, p < .001) and ShD group (F(4, 20) = 137.29, p < .001) showed (undesirable) decreases in MPT. This contrast between RP group and control and ShD groups suggests that RP group had more stable in MPT following ShD or dosing. The Welfare Assessment Framework (Hall, 2014) suggests that this reflects a lack of change in affective state.

The decrease in MPT between days 10–11 most likely reflects a change caused by ShD, as does the further decrease from day 10–15, with dosing. It was expected that events which caused a change in affective state would cause a change MPT and as the first ShD (for ShD and RP groups) and the first dose (for all groups) were two of the most aversive events during the study (being the least predictable, see 1.3), the change in MPT reflects this. RP group had similar MPT readings across all phases.

### 3.5 Time required to dose

The range of times taken for dosing was 46.02–1:43.04 s. Time to dose was compared between groups and while a same pattern of decreasing time across doses was seen, there was an significant effect of group (F(2, 60) = 2.317, p = 0.025) (Fig. 5), with ShD taking longer to dose than control (t(46) = 2.249, p = 0.029) and RP (t(46) = 2.054, p = 0.046). There was no difference between control and RP (p = 0.9).

## 4 General discussion

The aim of this study was to compare a number of variables between groups of dogs subject to oral gavage. We aimed to determine whether sham dosing was beneficial by comparing a sham dosed group to a control group. These groups were also compared to a third, Refined protocol group to determine whether the treatment and time investment could mitigate the negative effects of dosing by oral gavage. Our measures show a consistent welfare benefit of the Refined protocol to dogs, while Sham Dosing was shown to be not just an ineffective protocol for preparing dogs for dosing, but actually resulted in a decrease in welfare and increased time to dose, likely by priming for the aversive event.

### 4.1 Behaviour in the home pen and during dosing

Given that during Training phase, RP group were given several Refinements, but no aversive events, it is not surprising that dogs' welfare was higher than those with no interventions. The benefits of human interaction and training are well documented (Hennessy, Williams, Miller, Douglas, & Voith, 1998). Dogs progressed quickly through the training schedule (Appendix B) and the differences in behaviour between ShD and RP groups during dosing further illustrate the positive effects of the Refined protocol on welfare.

During Dosing phase, the differences in behaviour between control and ShD groups became more evident. In the home pen, control and ShD groups were spending more time with high and half-low posture, and less time with neutral posture and more with high or half-low posture than RP group. ShD group spent less time resting head down. Both groups exhibited more negative welfare indicators than RP group, although it appears that their responses were different. RP group spent less time sitting alert, at the front and with high posture, and more time resting head up or down and with neutral posture. Meanwhile, they also spent less time sitting alert, at the front, low tail wagging, with half-low or low posture and exhibiting fewer behavioural events than and more time tail wagging high and with neutral posture. Similarly, these differences in behaviour are seen during Training phase, which suggests that the training protocol prevented dosing having such a negative effect on RP group. ShD group exhibit more low tail wagging and low posture than other groups, even in the home pen, behaviours which are rarely seen in the home pen. This suggests a negative impact of sham dosing and dosing on their welfare.

This pattern of behaviour was further seen during dosing, with similar responses from both control and ShD groups and RP group. RP group spent less time sitting but resisting, struggling or ‘freezing’ but more time interacting with the handler and sitting relaxed. They also spent less time with high or low posture, or crouching and trembling, and more time with neutral posture. There were a number of differences in key behaviours between control and ShD groups which suggest that dosing had a more negative welfare impact on ShD group. ShD group made more escape attempts than control group and spent more time crouching. This suggests that the previous exposure to dosing protocol during sham dosing had not habituated ShD group to the procedure, but had rather primed an aversive response due to the lack of control and predictability surrounding sham dosing. In contrast, RP group had undergone sham dosing but with added aspects of control and predictability, and desensitisation rather than habituation, and this resulted in fewer negative changes in welfare compared to both control and ShD groups.

### 4.2 Welfare Monitoring Tool

**Fig. 3:**
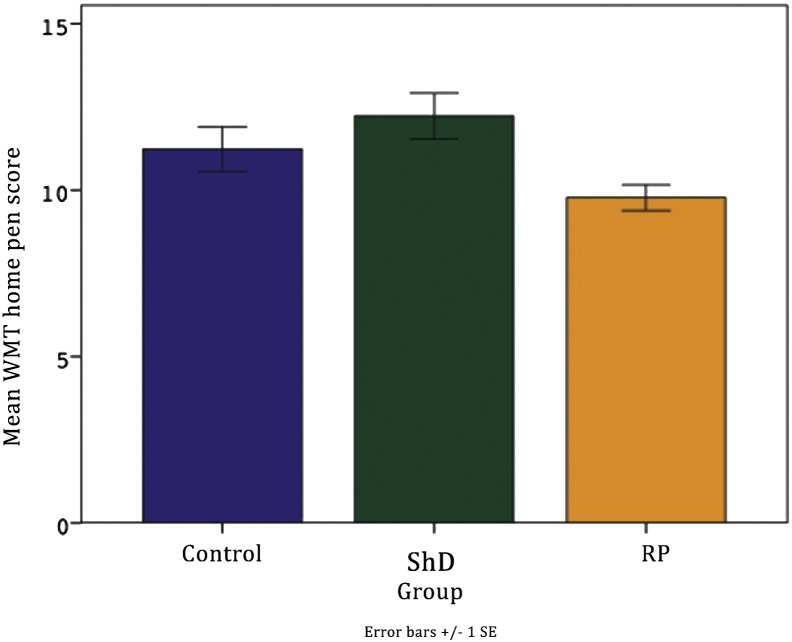
Mean total score by group across all phases. Lines show significant differences (p < 0.05).

**Fig. 4:**
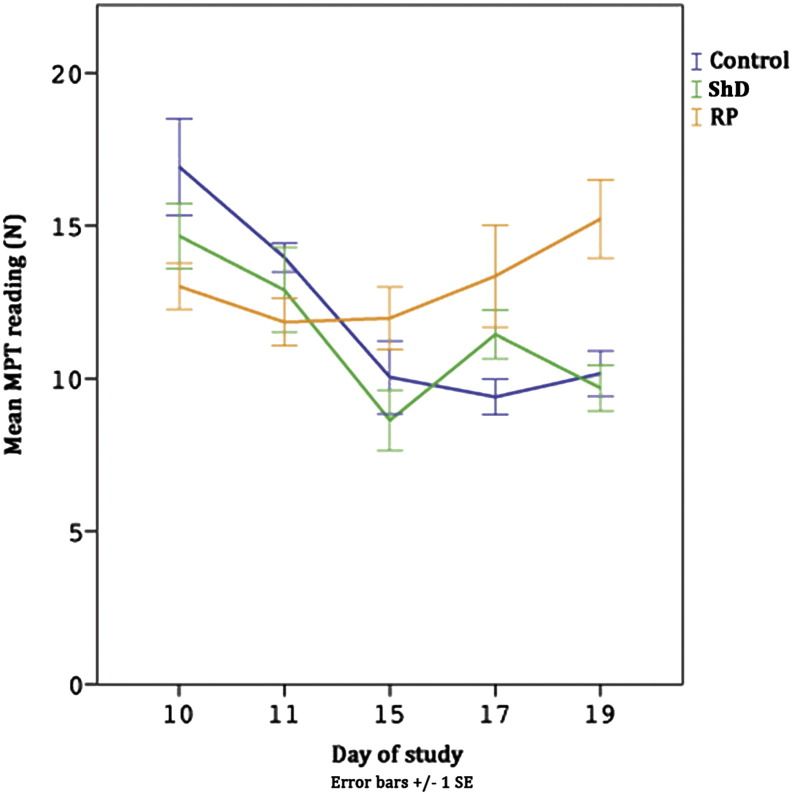
MPT readings over five days of dosing by group. Sham dosing occurred on days 10–11, while dosing occurred on days 11–19.

**Fig. 5:**
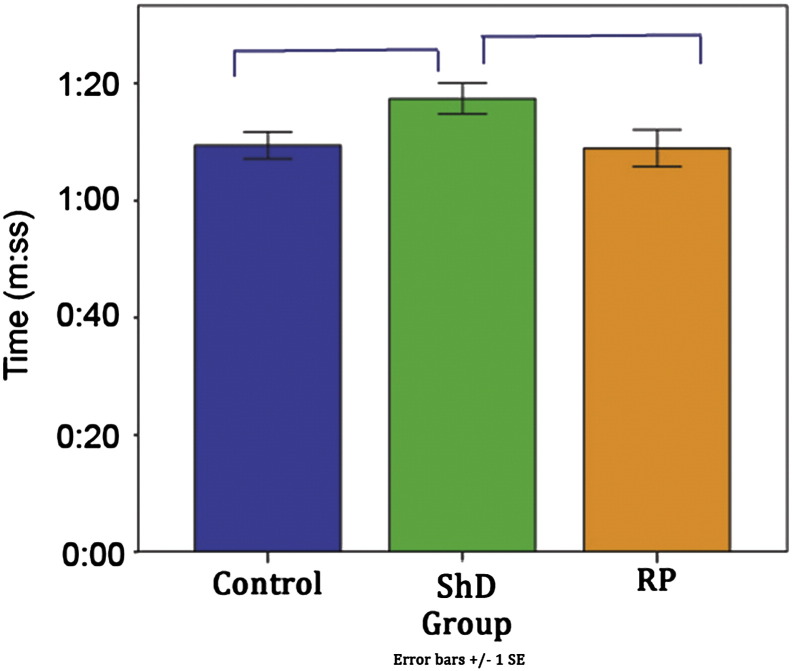
Mean time to dose by group.

The WMT agreed with other measures in a number of ways (Section 3.3). It was sensitive to the changes in behaviour which occurred in Dosing phase. The home pen score increased on the first day of dosing and decreased until the third day of dosing. During Dosing phase, both the dosing score and combined score decrease across the five doses, showing that dogs became increasingly habituated to the procedure across the week. When looking at the combined scores for all groups over time in Fig. 3, there is a trend towards ShD group having the highest scores, while RP group have the lowest scores, with control group falling between these. This agrees with other behavioural measures which shows ShD group find dosing more aversive than the other groups and RP group having the least negative response to dosing. Although between-group differences over time did not reach significance, RP group had significantly lower scores overall than ShD group, with the difference being marginally non-significant with control group. The WMT appears to be a useful method of detecting welfare changes which agrees with our other measures and can be implemented by staff.

### 4.3 Quality of data output

While it is clear that RP group has the best welfare, any Refined protocol must not interfere negatively with the quality of data obtained for a toxicology study. No clear pattern of food consumption was discernible and so it is concluded that food consumption is not affected by changing welfare, nor by implementing a Refined protocol. Body weight showed no changes over time, regardless of group (Section 3.4.1). As the interaction between food treats and dogs' food consumption and body weight is a commonly-cited argument against the use of PRT, these results support the use of a PRT protocol.

MPT dropped after the first day of sham dosing and again on the first day of dosing, indicating higher sensitivity to pressure, unsurprising as these were likely to be perceived as the two most aversive events during the study as the dogs were unlikely to be able to predict these events (Section 3.4.2 Bassett & Buchanan-Smith, 2007). Control and ShD groups showed decreases in MPT across time, while RP group showed no significant changes, suggesting stability in MPT and that dosing had less of an effect on them. This agrees with one of the aims of the study, that the training protocol should desensitise the dogs to dosing protocols and that this should mitigate the negative effects of dosing on welfare. Using the Welfare Assessment Framework (Hall, 2014), dogs in ShD group, and to some extent control group, are at risk of producing lower quality data and exhibiting greater undesirable within- and between-dog variation. Introducing such variation goes against the principles of ‘good science’ (Poole, 1997).

Our own research (Hall, 2014) and that of others (e.g. Everds et al., 2013), has shown a link between the welfare of laboratory-housed animals and the quality of the data obtained from their use. We believe that our findings warrant the investigation of the effects of Refinements to dosing protocols on key safety assessment parameters, including heart rate, blood pressure, clinical pathology and pathology end points.

### 4.4 Time required to dose

It was expected that RP group would be the quickest group to dose. Previous research (e.g. McKinley et al., 2003) has shown that PRT leads to increasing cooperation with the handler and technician, which in turn can decrease the length of time required to conduct procedures. While this did not prove to be the case, both control and RP groups were faster to dose than ShD (Section 3.5). This suggests that some factor caused the ShD group increased the time to dose them. Due to the increase in time spent ‘freezing’ while being dosed, and a clearly observable tension in the jaw while being dosed in several of the ShD dogs, it seems likely that this is the reason for the difference. Tension makes it difficult to open the mouth or insert the gavage tube. While the differences in time may appear subtle between control and RP (1:09 and 1:08 respectively) and ShD (1:17) groups, it should be considered as a factor when weighing up the benefits of a Refined protocol. Any increase in time to dose per dog as a result of ‘freezing’ behaviour is not desirable and further supports the conclusion that a non-sham dosing or Refined protocol is of greater benefit than a sham dosing protocol. The significantly faster dosing time for the RP group over the ShD group should be taken into consideration when implementing pre-study training protocols.

## 5 Conclusions

The data presented here suggest that the Refinements to oral gavage had a benefit for welfare. Dogs in RP group spent more time interacting with the handler and environment, and less time freezing, a behaviour which may often be mistaken for co-operation. Freezing is often the result of pseudo-habituation and the greater time required to dose ShD group dogs shows sham dosing to be an inefficient training method. Behaviour in the home pen also showed that dosing had less impact on welfare overall when compared to the other groups. Many of the positive changes seen during the Training phase were maintained through the Dosing phase and there was also a lack of change in MPT, suggesting a lesser impact of dosing on affective state, or at least sensitivity to mechanical pressure, which seemed to increase in the other groups following dosing. The technicians' WMT appears to be a useful way of monitoring welfare for staff, although it requires some further work to achieve agreement with other measures. The technician reported that the benefits of hourly monitoring of the dogs included increased familiarisation with the technician's presence (as demonstrated by the dogs not responding to the technician entering the room), increased familiarity with individual dogs' patterns of behaviour, and opportunities to observe natural behaviour, rather than a behavioural response to the technician's presence. The ability to closely monitor changes in behaviour is crucial to picking up subtle side-effects in toxicity testing and the use of the WMT encourages the technicians to identify individual dogs and recognise their normal behaviour.

Our previous research (Hall, 2014) found that dogs which are less susceptible to changes in welfare following aversive events also provide higher quality cardiovascular data. Given the results of this study, it is recommended that if it is not possible to provide an adequate pre-study training protocol that sham dosing not be substituted in its place. It is highly recommended that a Refined protocol for dosing by oral gavage like the one described in this study be followed to maximise welfare and data quality.

## Figures and Tables

**Table B.10:** PRT session outlines.

Session 1	Session 2
GOAL: Calm, relaxed removal from pen to procedure pod	GOAL: Sitting on-table behaviour
PROTOCOL: Reinforcer given for calm behaviour on procedure table	PROTOCOL: Sitting behaviour shaped using reinforcer

Session 3	Session 4

GOAL: Dog is relaxed while gently restrained by handler in seated position	GOAL: Dog is relaxed while restrained in the presence of technician
PROTOCOL: Sitting behaviour maintained, dog restrained by handler	PROTOCOL: Dog restrained by handler and sham dosing protocol mimicked

**Table B.11:** PRT training stages.

Stage	Description
A	Doesn't accept treats (excited or nervous)
B	Accepts treats from handler
C	Calm and relaxed on table
D	Attempts sitting behaviour
E	Shows brief sits
F	Maintains a longer sit
G	Sits well and tolerates gentle restraint

**Table C.12:** Locations within the home pen.

Location	Description
Front	Within the front half of the pen
Back	Within the rear half of the pen
Barrier	At the hatch in the barrier between two adjoining pens

**Table C.13:** Behavioural measures of positive welfare in the home pen.

Behaviour	Description	Source
Resting head up	Sitting or lying, not apparently asleep but not orientated towards any stimulus	Beerda, Schilder, Van Hooff, De Vries, & Mol (1998); Haverbeke, Laporte, Depiereux, Giffroy, & Diederich (2008)
Resting head down	Lying, may be apparently asleep, not orientated towards any stimulus	Beerda et al. (1998); Hubrecht, Serpell, & Poole (1992); Spangenberg, Björklund, & Dahlborn (2006); Haverbeke et al. (2008)
Interact with environment	Sniffing or investigating pen or objects	Beerda et al. (1998)
Amicable	Lick, play, allogroom dog, often with tail wag	Hubrecht et al. (1992); Spangenberg et al. (2006)
Solicit play	Bow, metaplay	Hubrecht et al. (1992); Hubrecht (1993); Spangenberg et al. (2006)
Play (self)	Usually involving toys or other objects	
Play (social)	Bouncing gait, play face, wrestle, play chase	Hubrecht et al. (1992); Hubrecht (1993); Spangenberg et al. (2006); Horowitz (2002)
Calm locomotion	Walk, 4 beat gait and 3 ft on the ground at any one time	Overall (2014)

**Figure:**
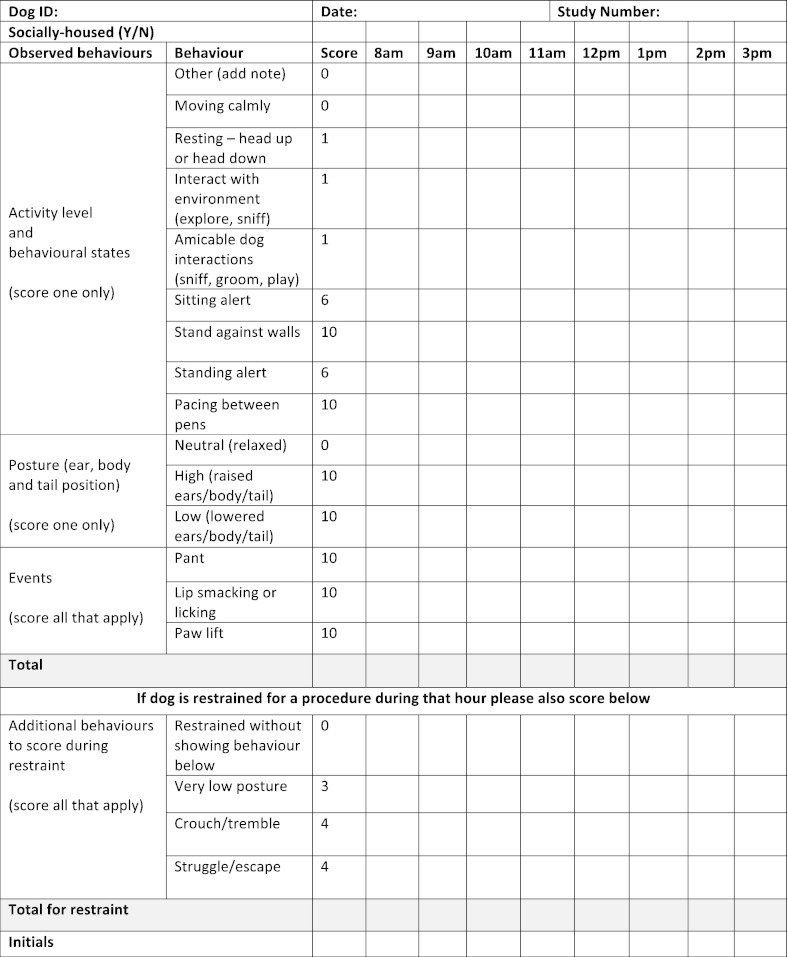


**Table C.14:** Behavioural measures of negative welfare in the home pen.

Behaviour	Description	Source
Stand against walls	Stands on hind legs with forelegs against wall	Beerda et al. (1998); Hubrecht et al. (1992), Hubrecht (1993); Beerda, Schilder, Van Hooff, De Vries & Mol (1999); Haverbeke et al. (2008); Spangenberg et al. (2006)
Circling	Repetitive movement around pen	Beerda et al. (1998); Hubrecht et al. (1992); Hubrecht (1993); Beerda et al. (1999); Haverbeke et al. (2008); Spangenberg et al. (2006)
Pace	Repetitive pacing, usually along a boundary	Hubrecht et al. (1992); Hubrecht (1993); Haverbeke et al. (2008)
Social pace	Repetitive pacing, in parallel with a dog on other side of boundary	Hubrecht et al. (1992); Hubrecht (1993)
Sit alert	Dog orientated towards stimulus while in a sitting position	Ley, Bennett, & Coleman (2008)
Stand alert	Dog orientated towards stimulus while in a standing position, usually accompanied by high posture	Ley et al. (2008)
Rapid locomotion	Trot, 2 beat gait, diagonally opposite legs move together	Overall (2014)

**Table C.15:** Postural measures of welfare in the home pen.

Behaviour	Description	Source
High	Breed specific posture as shown under neutral conditions, with the addition of high tail, head and ear position	Beerda et al. (1998); Beerda et al. (1999)
Neutral	Breed specific posture as shown under neutral conditions	Beerda et al. (1998); Overall (2014)
Half-low	Two features from: low position of tail, backwards bending of ears, bent legs	Beerda et al. (1998); Beerda et al. 1999); Haverbeke et al. (2008)
Low	As above, all three features present	Beerda et al. (1998); Beerda et al. (1999); Haverbeke et al. (2008)
Very low	As above, with body close to ground	Beerda et al. (1998); Haverbeke et al. (2008)
Tail wag high	Repetitive movements with the tail held high	Beerda et al. (1998); Beerda et al. (1999); Haverbeke et al. (2008); Normando, Corain, Salvadoretti, Meers, & Valsecchi (2009)
Tail wag low	Repetitive movements with the tail held low	Beerda et al. (1998); Beerda et al. (1999); Haverbeke et al. (2008); Normando et al. (2009)

**Table C.16:** Behavioural events indicating negative welfare in the home pen.

Behaviour	Description	Source
Oral behaviours	Includes tongue out, snout licking, swallowing, lip smacking	Beerda et al. (1998); Beerda et al. (1999); Haverbeke et al. (2008)
Paw lift	Sudden raising of one limb, usually foreleg, and usually in response to stimulus	Beerda et al. (1998); Beerda et al. (1999); Haverbeke et al. (2008); Spangenberg et al. (2006);Stephen & Ledger (2005)

**Table C.17:** Other behavioural measures in the home pen.

Behaviour	Description	Source
Crouch	Bent legs, body lowered towards ground	Beerda et al. (1998)
Tremble	Clear shivering of the body	Beerda et al. (1998)

**Table C.18:** Additional behavioural measures for challenges.

Behaviour	Description	Source
Struggle	Dog attempts to avoid restraint and/or human	McGreevy, Starling, Branson, Cobb, & Calnon (2012)
Escape attempts	All occurrences of attempts to leave table	

**Table 1:** Treatment delivered to each of three groups.

Group	Control	ShD	RP
Condition	Control	Sham dosing	Refined protocols

Treatment			
Health check	Once weekly in all phases	Once weekly in all phases	Once weekly in all phases
Training sessions	None	None	4 × in Training phase
Modifications to handling	None	None	All phases
Predictive signal for dosing	None	None	All phases
Sham dosing	None	2 × in ShD phase	2 × in ShD phase (Refined technique)
Vehicle-only dosing	Daily in Dose phase	Daily in Dose phase	Daily in Dose phase

**Table 2:** Results of ANOVAs between groups during Training phase.

Behaviour	F(2, 93)	p	Findings
Resting head down	7.765	0.014	RP > C, ShD
Sit alert	5.762	0.004	C, ShD > RP
High posture	4.434	0.015	ShD > C, RP
Neutral posture	5.753	0.004	C, RP > ShD

C, Control; ShD, Sham dosing; RP, Refined protocols.

**Table 3:** Results of Kruskal–Wallis tests between groups during Training phase.

Behaviour	*χ*^2^(2)	p	Findings
Back	6.857	0.032	RP > C, ShD
Half-low posture	8.801	0.012	C > RP

C, Control; ShD, Sham dosing; RP, Refined protocols.

**Table 4:** Results of ANOVAs between groups during Dosing phase.

Behaviour	F(2, 165)	p	Findings
Sitting alert	22.616	< 0.001	C, ShD > RP
High posture	35.338	< 0.001	ShD > C, RP
Neutral posture	26.150	< 0.001	RP, C > ShD
Resting head up	5.746	0.004	RP > ShD

C, Control; ShD, Sham dosing; RP, Refined protocols.

**Table 5:** Results of Kruskal–Wallis tests showing between-group differences during Dosing phase.

Behaviour	*χ*^2^(2)	p	Findings
Front	7.677	.022	ShD > RP
Resting head down	15.276	< 0.001	C, RP > ShD
Standing alert	22.918	0.022	ShD > RP
Half-low posture	61.358	< 0.001	C, ShD > RP
Low posture	6.067	0.048	ShD > C, RP
Paw lifts	29.508	< 0.001	C > ShD, RP
Behavioural events	18.836	< 0.001	C, ShD > RP

C, Control; ShD, Sham dosing; RP, Refined protocols.

**Table 6:** Results of ANOVAs showing effects of phase on home pen behaviour.

Behaviour	F(2, 338)	p	Findings
High posture	4.154	0.012	Dosing > Training
Neutral posture	74.16	< 0.001	Training > Dosing
Play	5.085	0.007	Training, ShD > Dosing

ShD, Sham dosing.

**Table 7:** Results of Kruskal–Wallis tests showing effects of phase on home pen behaviour.

Behaviour	*χ*^2^(2)	p	Findings
Half-low posture	17.160	< 0.001	Increase: Training < ShD < Dosing
Paw lifts	6.291	0.043	Increase: Training < Dosing
Behavioural events	6.850	0.033	Increase: Training < ShD

ShD, Sham dosing.

**Table 8:** Results of ANOVAs showing effects of group behaviour during dosing.

Behaviour	F(2, 60)	p	Findings
Interact with handler	3.159	0.036	RP > C, ShD
Struggle	4.523	0.015	C, ShD > RP
Freeze	27.407	< 0.001	C, ShD > RP
Paw lifts	2.572	0.034	C, ShD > RP

C, Control; ShD, Sham dosing; RP, Refined protocols.

**Table 9:** Results of Kruskal–Wallis tests showing effects of group on behaviour during Dosing.

Behaviour	*χ*^2^(2)	p	Findings
Sit relaxed	42.751	< 0.001	RP > C, ShD
Stand	9.093	0.011	C > RP
High posture	9.755	0.008	C, ShD > RP
Low posture	29.910	< 0.001	C, ShD > RP
Neutral posture	32.986	< 0.001	RP > C, ShD
Crouch	16.461	< 0.001	ShD > C > RP
Tremble	12.731	0.002	C, ShD > RP
Escape attempts	7.847	0.020	ShD > C, RP

C, Control; ShD, Sham dosing; RP, Refined protocols.

## Appendix A Additional Refinements provided to RP group

### Appendix A.1 Predictability

In order to provide a reliable signal for dosing, and therefore a shorter period of arousal, a visual signal was provided individually to each dog two minutes before dosing. A piece of A4 card was fixed to the front of the pen showing white, and was flipped two minutes before dosing (or training periods) to show black. It was flipped again to white when the dog was returned to the pen following training or dosing. This was designed to provide a brief, (perceived) controlled period of increased arousal immediately before dosing, decreasing the length of time spent in undesirable behavioural and affective states.

### Appendix A.2 Handling and control

Scruffing and removing a dog from the home pen for dosing removes choice and a sense of control for the dog. To mitigate this, as recommended in Prescott et al. (2004), dogs were not scruffed, but were encouraged to approach the handler before lifting and removal from the pen.

### Appendix A.3 PRT and desensitisation

Dogs received four five-minute training sessions in Training phase. A programme of desensitisation using PRT was followed. A training schedule and the stages of training are provided in 6. A step-by-step guide to training for each of the stages is provided in supplementary material. The steps followed The target behaviour was calm sitting on the table during restraint. Vocal praise, calm touch and a food treat (Pedigree® Cheesy Bites) were used as reinforcers and were used both in the procedure pod and immediately upon return to the home pen.

## Appendix B Session outlines and stages of training for PRT

Table B.10 describes the goals and protocols for each of the four training sessions received by RP group dogs.

Table B.11 shows the stages (A–G) through which dogs progressed in Training phase. The overall goal of Training phase was to have all dogs displaying Stage G reliably before dosing begins.

## Appendix C Coding scheme

The following tables show the behaviours used in behavioural observations and in the technicians' Welfare Monitoring Tool.

(See Tables C.12–C.18.)

## Appendix D Technicians' Welfare Monitor Tool

The technicians' Welfare Monitoring Tool is presented below. The behaviours included were those which most were most strongly associated with positively- or negatively-valenced welfare in our Welfare Assessment Framework (Hall, 2014). A score of 0 was given to behaviours which had no clear welfare indication — calm moving and ‘other’ (to allow the technician to add in behaviours of interest which weren't included). Desirable behaviours were given a score of 1, while undesirable behaviours were given a score of 6 (moderately undesirable) or 10 (highly undesirable). Behavioural events and high or low posture were also given a score of 10. This score weighting meant that if a dog exhibited undesirable behaviours only once or twice throughout the day, scores would only be moderately increased, whereas dogs which exhibited undesirable scores throughout the day would have consistently high scores, in agreement with the Welfare Assessment Framework. This differentiates between dogs exhibiting transitory changes in behaviour from those with consistent exhibition of negative welfare indicators.

Score range each hour: 0–75.

Interpretation: 0–25 no concern; 26–40 continue to monitor behaviour, consider intervention if score does not decrease; > 40 welfare concern, intervention recommended if score consistently elevated.

Maximum score possible for restraint: 11. Score > 1 intervention recommended.
